# A kidney transplant patient on cyclosporine therapy presenting with dural venous sinus thrombosis: a case report

**DOI:** 10.1186/1757-1626-2-9139

**Published:** 2009-12-03

**Authors:** Senaka Rajapakse, Rosana Gnanajothy, Niroshan Lokunarangoda, Rushika Lanerolle

**Affiliations:** 1 Department of Clinical Medicine, Faculty of Medicine, University of Colombo,25, Kynsey Road, Colombo 08, Sri Lanka; 2 University Medical Unit, National Hospital Colombo, Regents Street, Colombo 08, Sri Lanka

## Abstract

Dural vein thrombosis is an important but rare cause of headache. Therapy with cyclosporine has been reported to result in thrombotic events. We report an unusual case of superior sagittal and transverse sinus thrombosis associated with cyclosporine therapy in a kidney transplant patient.

## Introduction

Dural vein thrombosis is an important but rare cause of headache. It is associated with a wide range of conditions. Therapy with cyclosporine has been reported to result in thrombotic events. We report an unusual case of superior sagittal and transverse sinus thrombosis associated with cyclosporine therapy in a kidney transplant patient.

## Case presentation

A 25-year-old Sri Lankan Sinhalese male post kidney transplant patient presented with frontal headache of insidious onset which worsened over 3 days, associated with vomiting and fever. Neurological examination was normal on admission, and over the next 12 hours he developed weakness of the left side of the body, and soon afterwards developed a left sided focal seizure with secondary generalization. There had been no previous episodes of similar headaches or seizures. He had had membrano-proliferative glomeulonephritis progressing to end-stage renal failure necessitating a live-related-donor kidney transplant 11 months ago, and had been well post transplant. He had been on immunosuppressive therapy post- transplant: prednisolone, and cyclosporine 200 mg twice daily for the first 5 months post transplant, later reduced to 200 mg mane and 100 mg nocte, which was the dose on admission. He had also developed diabetes 8 months ago, likely to be steroid induced. He had no past history of rheumatological or autoimmune disorder, and had had no previous episodes of venous or arterial thrombosis.

He had no features of cyclosporine toxicity such as tremors, hirsutism, or gum hyperplasia. Examination after the seizure showed increased reflexes, increased tone and reduced motor power to grade 4/5 on the left upper and lower limbs. Bilateral papilloedema was present. Cardiovascular and respiratory system examination was normal, and his blood pressure was normal throughout.

**Figure 1:**
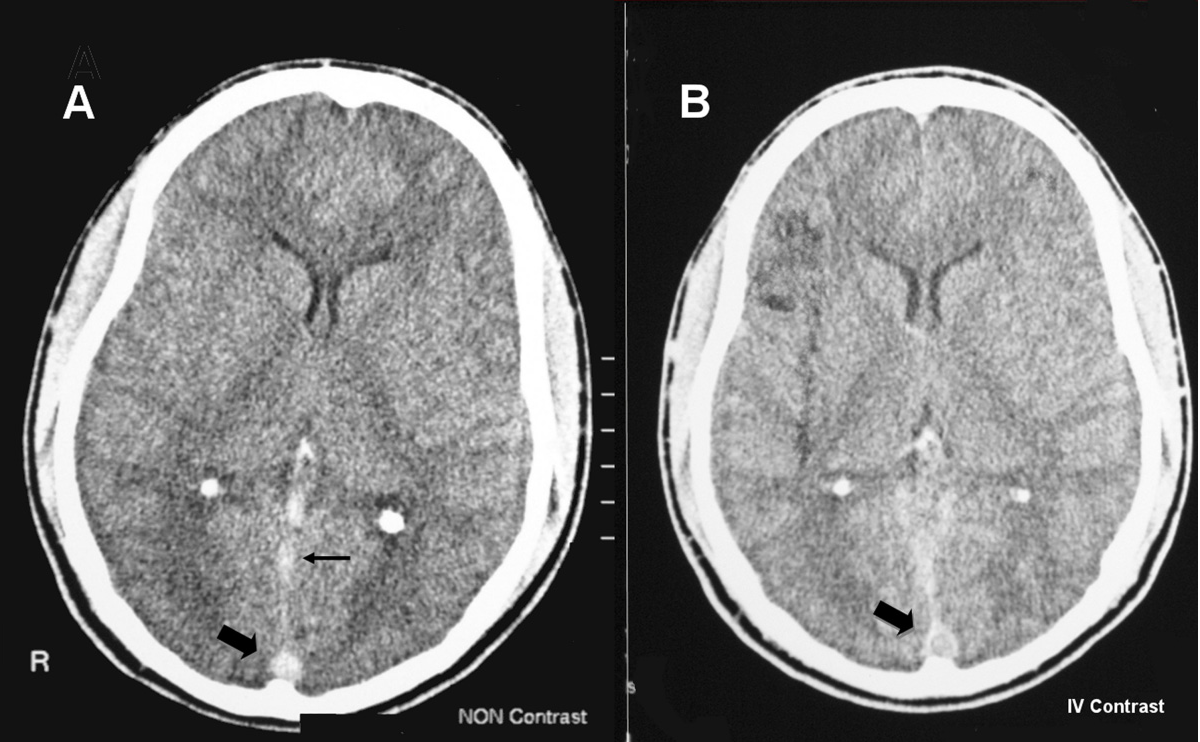
**A. Non contrast enhanced CT scan showing the dense delta sign (thick arrow), with hyperintensity of the sagittal sinus (thin arrow)**. **B**. Contrast enhanced CT scan showing the empty delta sign (thick arrow).

**Figure 2:**
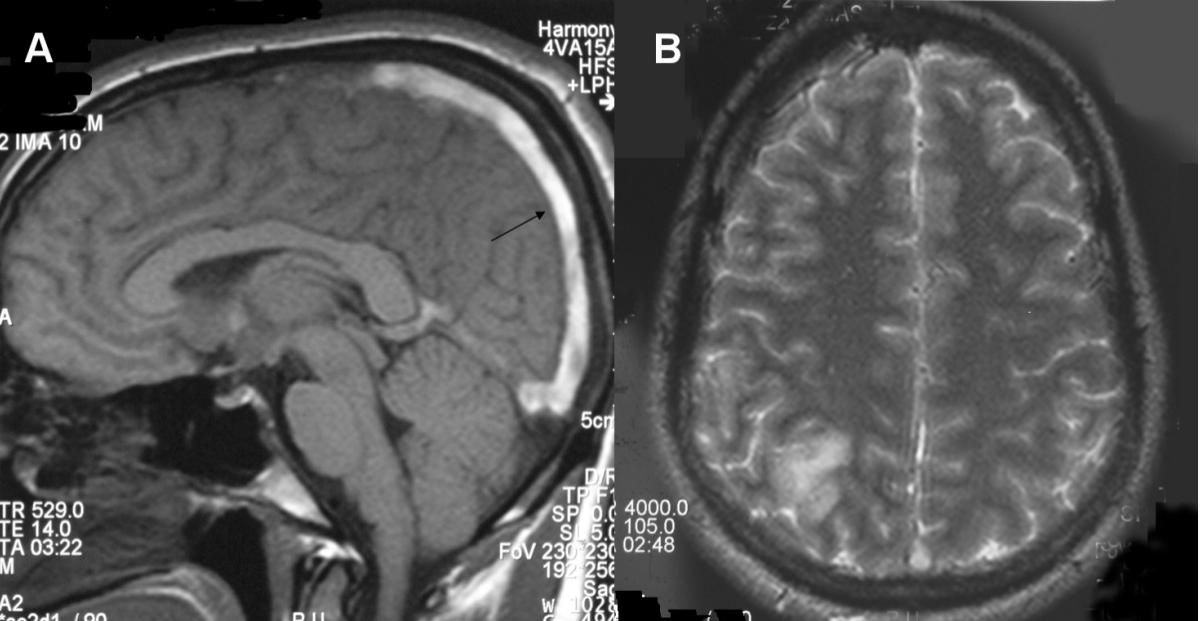
**A. T1 weighted MRI - The superior sagittal sinus shows a hyper-intense signal of the thrombus seen in place of low-signal flowing blood**. **B**. T2 weighted MRI showing venous infarct in right parieto-occipital region.

Full blood count showed a relative lymphocytosis and mild thrombocytopenia. His blood picture showed no evidence of microangiopathic haemolytic anaemia, and coagulation profile was normal. Cytomegalovirus studies (CMV PCR, CMV antigen test, CMV IgM antibodies) were negative. Renal function tests were normal, and urine analysis was clear, with no proteinuria. Serum albumin levels were normal. Cyclosporine levels (trough) were within therapeutic range. Cerebrospinal fluid examination, including gram stain and AFB smears, done on day 1 was normal; PCR for tuberculosis, and fungal and viral studies were negative, and CSF bacterial and fungal cultures showed no growth. A non-contrast-enhanced CT scan of the head was performed (Figure 1A); no signs of cerebral infarction or hemorrhage were seen, however cerebral oedema was present. The 'dense triangle sign' was observed, with hyper-intensity of the straight sinus, suggestive of superior sagittal sinus thrombosis. Contrast-enhanced CT scan of the head (Figure 1B) showed the classical sign of superior sagittal sinus thrombosis, namely the 'empty delta sign'. MRI brain confirmed the diagnosis (Figure 2A), and also revealed thrombosis of the right transverse sinus with a venous infarct of the right parieto-occipital region (Figure 2B). Haematological and biochemical screen for a prothrombotic tendency was normal.

The patient's cyclosporine dose was reduced from 300 mg to 200 mg daily. Prednisolone was continued without dose modification. Anticoagulation was commenced with low-molecular-weight-heparin and continued with warfarin. Our patient's neurological symptoms and signs recovered completely, and he was discharged for routine clinic follow-up

## Discussion

Dural venous thrombosis is an uncommon condition, and the hospital frequency of dural venous thrombosis is around 3-8 per 100000 population [1-3]. It is commoner in women [3], and affects younger individuals [2,3], compared with arterial infarcts which more commonly occur in older patients. The superior sagittal sinus is the commonest site [4], and partial thrombosis may account for up to 50% of these cases. Clinical presentations fall into four patterns; pseudotumour syndrome, cerebral infarction, haemorrhage or venous hypertension [1]. Headache is the commonest clinical feature, and is seen in around 90% of cases [1,4]. Focal neurological defects and impaired level of consciousness are seen in around 50% of patients; papilloedema and seizures also occur. Many causes have been described [2,4]; a prothrombotic haematologic disorder (antiphospholipid antibody syndrome, homocysteinuria, Factor V Leiden, protein C deficiency, paroxysmal nocturnal haemoglobinuria, essential thrombocythaemia, thrombrotic thrombocytopenic purpura), vasculitic disorders, sepsis (meningo-encephalitis, mastoiditis), trauma, severe dehydration, and certain drugs. Pregnancy, or the use of oral contraceptive drugs may be responsible in women. An aetiology is not identified in around 13% of patients [4].

CT scan of the head demonstrates dural venous thrombosis in about two thirds of cases [5], and is normal in the rest. In a non-contrast CT, the dense triangle sign is seen, i.e., hyperdensity with a triangular or round shape in the posterior part of the superior sagittal sinus caused by the venous thrombus [5,6]. On contrast-enhanced CT, the characteristic 'empty delta' sign is seen as a triangular pattern of contrast enhancement surrounding a central region without enhancement formed by the thrombus [7,8]. Indirect signs of thrombosis are frequently found- these include intense contrast enhancement of falx and tentorium, dilated transcerebral veins, small ventricles, and parenchymal abnormalities [5-8].

The thrombosed sinus may be difficult to identify on MRI scan during the acute stage of thrombosis, when the clot tends to be iso-intense with brain on T1-weighted images and hypo intense on T2-weighted images where it mimics a flow void. From 3 to 7 days after thrombosis, the clot becomes hyper intense on both T1- and T2-weighted images and is thus easier to identify [9]. The MRI was done on day 7 in our patient, and the clot is hypertense (Figure 2A).

Cyclosporine is known to increase the risk of thrombosis. The mechanism of this is complex. Cyclosporine increases platelet aggregation, and aggregability increases at higher cyclosporine levels [10,11]. Platelet thromboxane A2 release is also increased by cyclosporine, although there is conflicting evidence as to whether this is related to blood levels. The coagulation system is also affected by cyclosporine, and some of these changes favor thrombosis (elevated plasma fibrinogen levels, increased plasma viscosity). Cyclosporine is also known to result in haemolytic uraemic syndrome-thrombotic thrombocytopenic purpura (HUS-TTP) in transplant patients [12], and TTP can also result in thrombosis. Cyclosporine induced HUS-TTP in renal transplant patients is different from classical HUS-TTP. Thrombocytopenia and micro-angiopathic haemolytic anaemia, which are the essential diagnostic criteria in classical HUS-TTP, may be absent in cyclosporine induced HUS-TTP, and acute renal failure is the principal manifestation [13,14]. Sinus venous thrombosis has been reported in patients after bone marrow transplantation who were on cyclosporine, although TTP was not demonstrated in these patients [15]. Although mild thrombocytopenia was present, our patient had no evidence of microangiopathic haemolytic anaemia, nor was there any evidence of renal involvement resulting from micro-angiopathy, and cyclosporine induced HUS-TTP was hence unlikely.

Dural sinus thrombosis due to meningo-encephalitis predisposed to by immunosuppression was considered unlikely, as the cerebrospinal fluid examination was normal. No other coagulopathy could be identified. Although glomerulonephritis with proteinuria increases the risk of thrombosis, our patient had a stable functioning kidney transplant for many months, and had no proteinuria. Hypercoagulability associated with nephrotic syndrome was therefore not likely. Cyclosporine induced thrombosis, through a direct thrombotic effect, was the most likely cause for our patients thrombosis. In our patient, this took place with cyclosporine levels within therapeutic range. Anticoagulation with heparin is the most widely used treatment for dural vein thrombosis[4], and has been shown to be beneficial [16,17], although a meta-analysis failed to show statistical significance [18]. The overall prognosis for dural vein thrombosis is good [4], with complete recovery in 57%. The mortality is around 8%. This is the first report in the literature of dural sinus thrombosis caused by cyclosporine in a renal transplant patient.

## Abbreviations

CMV: cytomegalovirus; CT: computerized tomography; HUS-TTP: hemolytic uremic syndrome-thrombotic thrombocytopenic purpura; IgM: immunoglobulin M; MRI: magnetic resonance imaging; PCR: polymerase chain reaction.

## Consent

Written informed consent was obtained from the patient for publication of this case report and accompanying images. A copy of the written consent is available for review by the Editor-in-Chief of this journal.

## Competing interests

The authors declare that they have no competing interests.

## Authors' contributions

RG and NL interpreted the patient data regarding the clinical history, progression and investigations. SR analyzed the information, did the literature searches and wrote the manuscript. RG, NL, SR and RDL were involved in clinical care of the patient, and their discussions formed the basis of the case report. All authors read and approved the final manuscript.
